# Odds-ratio network for postoperative factors revealing differences in the 2-year longitudinal pattern of satisfaction between women and men after total knee arthroplasty

**DOI:** 10.1038/s41598-022-21541-5

**Published:** 2022-10-19

**Authors:** J. Gallo, E. Kriegova, M. Radvansky, M. Sloviak, M. Kudelka

**Affiliations:** 1 Department of Orthopedics, Faculty of Medicine and Dentistry, Palacky University Olomouc & University Hospital Olomouc, Hnevotinska 3, 775 15 Olomouc, Czech Republic; 2 Department of Immunology, Faculty of Medicine and Dentistry, Palacky University Olomouc & University Hospital Olomouc, Hnevotinska 3, 775 15 Olomouc, Czech Republic; 3 Department of Computer Science, Faculty of Electrical Engineering and Computer Science, VSB-Technical University of Ostrava, 17. Listopadu 2175/15, 708 00 Ostrava-Poruba, Czech Republic

**Keywords:** Data mining, Quality of life

## Abstract

Timely and accurate assessments of the factors influencing satisfaction, a key indicator of success in primary total knee arthroplasty (TKA), may help improve TKA outcomes. Here we performed the longitudinal trend analysis of relation between satisfaction and 12 postoperative factors, which positively or negatively influence the patient satisfaction 2 years after TKA. In a real-world registry cohort (women/men: 1121/650), we showed similarities and differences between women and men in the contribution of postoperative factors to satisfaction 2 years after TKA as assessed by odds-ratio-similarity network. In men, the strongest negative factors were pain and complications, followed by mechanical problems. In women, the strongest negative factors were the pain and knee instability, followed by other mechanical problems, complications and low levels of sports activity. In both sexes, physical activity and the Knee Society Score (general and functional) influenced positively satisfaction; long-distance walking was associated with satisfaction only in women. A trend analysis revealed a reduction in the strength of satisfaction-related factors over 2 years of check-ups, particularly in women. Our study demonstrates that the key check-up for assessing the evolution of satisfaction in the 2 years after TKA was at 3 months in both sexes.

## Introduction

The goal of total knee arthroplasty (TKA) is to relieve pain, improve the functional abilities of the affected knee and thereby increase the health-related quality of life. Although primary TKA is one of the most successful orthopaedic procedures, only 72–89% of patients are satisfied with the results^1–5^. Given the expected increase in the number of TKA procedures, which is projected to reach 1.26 million per year by 2030 in the US alone^6^, a deeper understanding of the factors contributing to patients not achieving full satisfaction is required. This is an indicator that can negatively affect not only the patient's quality of life but also the surgeon's reputation.

Satisfaction is the quality or state of being satisfied with TKA outcomes in terms of the needs or wishes of the patient being fulfilled; not achieving full satisfaction may lead to feelings of displeasure or disappointment. However, the measurement of satisfaction is neither trivial nor uniquely related to TKA because it also integrates a number of health factors, non-health experiences and feelings into a single number or a particular response^7^. Satisfaction evaluation was introduced into TKA clinical practice in the 1990s by the Oxford Group as part of patient-reported outcome measures (PROMs)^8^. PROMs are questionnaires completed by patients to assess the effects of disease and/or treatment on symptoms, functioning and health-related quality of life from the patient’s perspective^9^. In this regard, the satisfaction question ‘How satisfied are you with your knee replacement?’ was first introduced in 2000 as a specific endpoint in the Swedish Knee Arthroplasty Registry^10^.

Different studies consider satisfaction from different perspectives, often providing inconsistent results; differences may result from a lack of homogeneity between studies, different measures of satisfaction and evaluations being conducted at different time points after surgery^11^. A systematic review summarising preoperative, intraoperative and postoperative factors revealed that the most common factors associated with satisfaction after TKA were patients' preoperative expectations, the degree of improvement in knee function and pain relief after surgery^1^. Recent studies have demonstrated that satisfaction evolves over time, and factors that influence satisfaction can be identified soon after, and even before, surgery^12,13^.

To gain a deeper insight into the longitudinal trends in evolving satisfaction in relation to the many TKA-related postoperative factors, we analysed patient data from the 2 years after TKA, obtained from a local joint replacement registry^14^. The impact of the most informative factors and their combinations on satisfaction was calculated for both sexes because emerging evidence suggests that women and men have different patterns of satisfaction^15^. Uncovering factors that influence satisfaction could improve patient outcomes and the strategies employed to increase satisfaction.

## Material and methods

### Patients

The unselected study cohort from a single centre’s register of joint replacements at the University Hospital included 1771 patients (1121 women/650 men) who underwent primary TKA surgery between October 2010 and January 2019 at this single tertiary orthopaedic centre. For details on surgery, postoperative care, and distribution of values of particular parameters, see Gallo et al.^14^.

The data in the registry were collected directly from the patients by a trained employee; clinical and/or radiographic data were provided by the attending surgeons or the surgeons administering the register, as reported previously^14^. Briefly, the data were continuously recorded in a Microsoft Access database, starting at the patients’ admission a day before the TKA surgery and then covering the intraoperative and early/late postoperative period. Patients underwent regular check-ups at 3-, 6-, 12- and 24-months postoperatively^14^.

Satisfaction was evaluated during each check-up through the patient’s response to the question ‘How satisfied are you with your knee replacement?’ using a 5-point visual equidistant (interval-level) Likert scale (4 = ‘extremely satisfied’, 3 = ‘very satisfied’, 2 = ‘moderately satisfied’, 1 = ‘a little satisfied’ and 0 = ‘not at all satisfied’).

Twelve preoperative and postoperative parameters were then investigated at each check-up: pain using the visual analogue scale (VAS), general Knee Society score (KSS), functional KSS^16^, ability to engage in long-distance walking (> 1000 m), University of California (UCLA) activity score^17^, sports activity (regular fast walking, cycling or swimming), mechanical problems (instability, foreign body sensation and clunk syndrome), anterior knee pain (AKP), complications (wound healing disturbances or early reoperations) and body mass index (BMI) (Table 1).

**Table 1:** Personal and clinical preoperative parameters in enrolled TKA patients subgrouped according to sex (1,121 women/650 men).

Dichotomized factors^*^	Value	Distribution of factors associated with satisfaction at particular check-ups in women/men (%)				
		Pre operative	3 months after TKA	6 months after TKA	12 months after TKA	24 months after TKA
Age (≤ 70/ > 70)	< 70	51/59				
Osteoarthritis (primary/secondary)	Secondary	15/20				
Kellgren-Lawrence (II–III C/III D–IV D)	III D–IV D	27/28				
BMI [kgm^−2^] (≤ 30/ > 30)	> 30	54/35	54/35	53/38	50/43	51/50
Physical activity UCLA (no-low, 1–3/middle-high, > 4)	middle-high	70/81	34/43	56/60	70/83	76/79
Sports activity (no/active)	Active	5/16	6/9	9/17	15/14	16/17
Walking distance (≤ 1000 m/ > 1000 m)	> 1000 m	15/28	80/87	93/93	96/97	97/96
General KSS (≤ 70/ > 70)	> 70	5/8	91/94	93/92	97/95	98/94
Functional KSS (≤ 70/ > 70)	> 70	2/7	13/20	28/39	44/63	58/54
VAS pain (low-middle/high)	High	34/34	2/2	2/1	2/3	2/3
Early post-operative complications (no/yes)	Yes		3/6	4/4	9/11	5/8
AKP (no/yes)	Yes		15/17	14/19	14/15	10/14
Clunk syndrome (no/yes)	Yes		14/17	13/15	18/13	11/12
Instability (no/yes)	Yes		32/29	24/15	23/22	18/21
Foreign body sensation (no/yes)	Yes		18/16	20/14	18/14	13/9

All factors were dichotomized (according to the values shown in parentheses); the distribution of factors associated with satisfaction at particular check-ups is shown.

n, number; AKP, anterior knee pain; BMI, body mass index; TKA, total knee arthroplasty; VAS, visual analogue scale; KSS, Knee Society score; UCLA, University of California.

*Dichotomization was performed considering the distribution of parameter values in the studied cohort and their clinical relevance (see Gallo et al.^14^).

The use of the clinical register of joint replacements was approved by the hospital management, and its administration was regulated by the amended ethical and legal protocol. All patients provided written informed consent about the usage of data for this study, which was performed in accordance with the Helsinki Declaration and approved by the ethics committee of the University Hospital and Palacký University Olomouc.

### Trend analysis of the relationship between satisfaction and postoperative factors

To assess trends (including 95% confidence intervals (CI)) in evolving satisfaction in relation to a particulate set of factors, an overall satisfaction questionnaire in the 2 years after TKA was introduced. First, the assumption that individual patients report similar levels of satisfaction at different check-up points was verified. For individual patients, overall satisfaction was determined based on the average satisfaction of the patient's self-reported satisfaction using the 5-point Likert scale at particular check-ups. Finally, the threshold for overall satisfaction was set at a first quartile value of overall satisfaction within the group of women and men.

### Odds-ratio-similarity network analysis of positive and negative factors associated with overall satisfaction

The relationship between factors and overall satisfaction was assessed and visualised using odds-ratio (OR)-similarity networks, with women and men assessed separately. The networks were constructed using the LRNet algorithm^18^ for the 12 aforementioned postoperative factors describing the likelihood of satisfaction if the factor was present in the patient´s group. In the visualised networks, the vertices represented factors and the ties of different strengths between vertices represented locally significant similarities between pairs of factors in relation to satisfaction.

To construct the networks, a vector representation of the evolution of the relationship between individual factors and overall satisfaction was used. Therefore, for each factor, four OR values from different check-ups (3-, 6-, 12- and 24-months) were calculated for groups of women and men; these four ordered OR values were used as vectors. With each vector representation, the similarity between each pair of vectors could be measured precisely, and the similarity between two vectors could be interpreted as a similar evolution of the relationship between overall satisfaction and the two factors. Moreover, similarity defined in this manner could be used to cluster vertices in the networks and thus identify groups of factors with a similar evolution.

## Results

### Overall satisfaction in women and men

To calculate overall satisfaction, we first verified that individual patients reported similar levels of satisfaction at different check-ups. On average, the difference between the maximum and minimum value for a single patient was 0.64 on the visual equidistant Likert scale, which is less than the distance between neighbouring values. This allowed us to calculate overall satisfaction for patients for whom satisfaction responses were available at only two check-ups. The average satisfaction for individual check-ups in women/men was 2.97/2.92 (3 months), 2.87/2.82 (6 months), 2.92/2.86 (12 months) and 2.91/2.76 (24 months), and the overall average satisfaction across the whole dataset was 2.91/2.84.

The cut-off for overall satisfaction in women/men was set at a first quartile value of 1.22/1.17, and the 25% of patients with overall satisfaction levels lower than this cut-off were considered ‘not fully satisfied’; the rest, with overall satisfaction levels higher than this cut-off, were considered ‘satisfied’. Thus, the patients who were not fully satisfied were those who predominantly reported themselves to be ‘not at all satisfied’ (0 on the Likert scale) or ‘a little satisfied’ (1 on the Likert scale), including those who rarely reported themselves to be ‘moderately satisfied’ or above at any of the check-ups, and the satisfied patients were those who predominantly reported themselves to be ‘moderately satisfied’, ‘very much satisfied’ or ‘extremely satisfied’ at the check-ups. The analysis was conducted based on separate groups of women and men, not on individual patients.

### Analysis of preoperative factors and satisfaction with TKA

In women, the preoperative factors of BMI, general and functional KSS, VAS pain score, UCLA activity and long-distance walking ability were not associated with postoperative satisfaction. The only preoperative factor associated with lower overall satisfaction levels after TKA (0.55, 95%CI 0.32–0.96, p = 0.033) was sports activity (fast walking, cycling and swimming) (Table 2).

**Table 2:** Contribution of the studied factors to overall postoperative satisfaction within 2 years of TKA in (A) women and (B) men at particular check-ups.

	Preoperative	3 months	6 months	12 months	24 months
**(A) Women**					
BMI > 30	1.06 (0.80–1.41)	0.45 (0.10–1.91)	0.66 (0.30–1.44)	1.11 (0.45–2.75)	0.63 (0.34–1.16)
Activity	1.02 (0.75–1.39)	**2.71 (1.51–4.85)**	**3.44 (1.63–7.26)**	**4.61 (1.75–12.11)**	1.61 (0.45–5.84)
Sports activity	**0.55 (0.32–0.96)**	0.70 (0.20–2.50)	0.61 (0.22–1.73)	**0.42 (0.18–0.97)**	0.79 (0.40–1.54)
Walking distance	1.19 (0.80–1.79)	**2.40 (1.08–5.32)**	**3.14 (1.13–8.75)**	**4.31 (1.22–15.20)**	0.94 (0.18–4.76)
General KSS	1.55 (0.72–3.35)	**2.92 (2.39–6.13)**	**5.41 (2.45–11.94)**	2.82 (0.73–10.82)	1.44 (0.34–6.15)
Functional KSS	1.92 (0.56–6.50)	2.89 (0.88–9.54)	**3.44 (1.43–8.29)**	**2.62 (1.29–5.33)**	0.79 (0.49–1.28)
VAS pain	0.98 (0.76–1.33)	**0.06 (0.02–0.26)**	**0.26 (0.09–0.73)**	**0.10 (0.02–0.53)**	0.69 (0.15–2.81)
Complications	1.01 (0.76–1.34)	**0.52** (0.14–1.89)	**0.61** (0.17–2.20)	**0.14 (0.06–0.29)**	0.54 (0.19–1.56)
AKP	–	**0.29 (0.13–0.59)**	**0.34 (0.14–0.81)**	0.49 (0.19–1.29)	1.07 (0.43–2.63)
Clunk syndrome	–	**0.51** (0.22–1.14)	**0.41 (0.18–0.95)**	**0.31 (0.14–0.72)**	1.37 (0.57–3.30)
Patellar instability	–	**0.25 (0.13–0.49)**	**0.43 (0.22–0.85)**	**0.38 (0.18–0.80)**	1.03 (0.55–1.94)
Foreign body sensation	–	**0.38 (0.19–0.73)**	**0.54** (0.26–1.13)	**0.49** (0.22–1.08)	**0.89** (0.44–1.79)
**(B) Men**					
BMI > 30	1.06 (0.70–1.61)	1.10 (0.09–13.54)	0.62 (0.24–1.59)	1.06 (0.32–3.51)	0.72 (0.29–1.81)
Activity	**1.68 (1.04–2.71)**	**2.82** (0.94–8.44)	**1.85** (0.64–5.38)	**5.65 (1.49–21.52)**	**5.00 (1.27–19.66)**
Sports activity	**0.80** (0.47–1.38)	2.18 (0.12–38.72)	0.43 (0.17–1.09)	0.56 (0.17–1.84)	0.71 (0.25–1.99)
Walking distance	0.94 (0.60–1.48)	0.29 (0.02–5.01)	1.90 (0.48–7.54)	2.10 (0.22–19.80)	6.89 (1.09–43.69)
General KSS	1.39 (0.59–3.05)	0.99 (0.13–7.92)	**6.73 (2.51–18.05)**	**4.22 (1.18–15.10)**	3.28 (0.94–11.42)
Functional KSS	0.88 (0.40–1.96)	**4.27** (0.56–32.78)	**2.13** (0.96–4.75)	**2.41 (1.07–5.43)**	**2.13 (1.02–4.43)**
VAS pain	0.80 (0.51–1.28)	**0.05 (0.01–0.36)**	**0.09 (0.03–0.36)**	0.18 (0.03–1.12)	0.36 (0.06–2.22)
Complications	1.07 (0.71–1.62)	**0.26 (0.07–1.00)**	**0.23 (0.06–0.87)**	**0.14 (0.05–0.35)**	0.61 (0.15–2.47)
AKP	–	0.38 (0.08–1.25)	**0.27 (0.09–0.80)**	**0.26 (0.08–0.83)**	0.53 (0.15–1.84)
Clunk syndrome	–	**0.32** (0.09–1.17)	**0.56** (0.20–1.55)	**0.20 (0.07–0.57)**	1.13 (0.30–4.25)
Patellar instability	–	**0.22 (0.06–0.77)**	**0.32 (0.13–0.79)**	**0.32 (0.12–0.83)**	0.69 (0.29–1.64)
Foreign body sensation	–	**0.36** (0.11–1.27)	**0.23 (0.09–0.56)**	**0.36** (0.13–1.04)	**0.51** (0.17–1.58)

Values represent odds ratios (OR; 95%CI, low–high value). OR values above 1 are associated with satisfaction; values below 1 are associated with not being fully satisfied. Values with ORs associated with satisfaction at particular check-ups are marked in bold.

Despite larger CIs resulting from greater data diversity or fewer patients per check-up examination, time-dependent trends for analysed factors are evident (in bold).

In men, the preoperative factors of BMI, KSS, VAS pain score and walking distance did not influence satisfaction after TKA, whereas men who reported sports activity as a preoperative factor were not fully satisfied postoperatively (0.80, 95%CI 0.48–1.38, p = 0.045) (Table 2). In the ‘not fully satisfied’ group of men, those of a younger age (< 70 years) had greater representation. Men who were physically active before surgery had a higher likelihood of being satisfied (1.68, 95%CI 1.04–2.71, p = 0.033).

### Longitudinal trend analysis of the relationship between postoperative factors and overall satisfaction

#### Postoperative knee function and satisfaction

**Figure 1:**
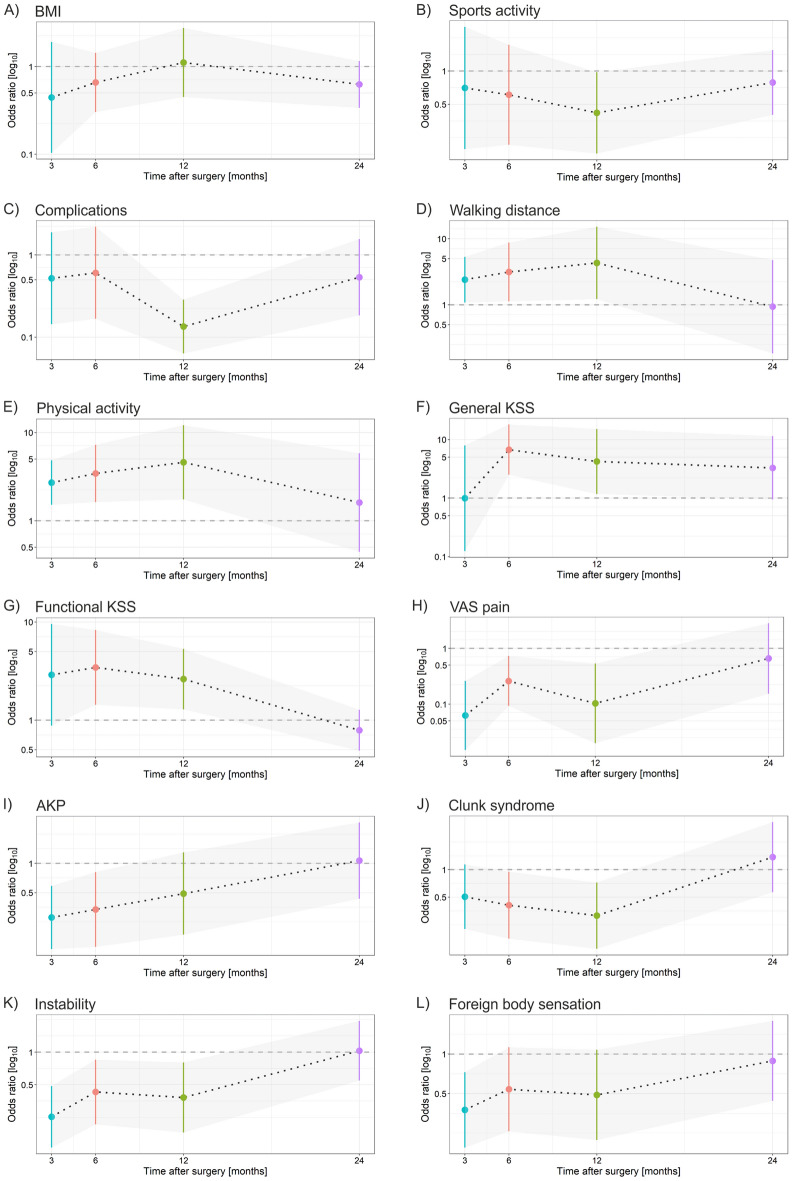
Contribution of postoperative factors to the likelihood of satisfaction in women in the 2 years after TKA. (**A**) BMI, (**B**) sports activity, (**C**) complications, (**D**) walking distance, (**E**) physical activity, (**F**) general KSS, (**G**) functional KSS, (**H**) VAS pain, (**I**) AKP, (**J**) clunk syndrome, (**I**) instability, (**K**) foreign body sensation. Odds-ratio (marked by dots) values above 1 are associated with satisfaction; values below 1 are associated with not being fully satisfied. The bars represent odds ratios (95%CI, low–high value).

We then evaluated postoperative knee function according to the general and functional KSS. In women, the faster the general KSS (> 70) increased postoperatively, the higher the likelihood of satisfaction compared with those with a general KSS below 70 (Fig. 1; Table 2). The functional KSS (> 70) began to indicate an increasing likelihood of overall satisfaction at the 6- and 12-month check-ups. By contrast, the functional KSS value reported at 3 months postoperatively did not influence overall satisfaction with the surgery (Fig. 1).

**Figure 2:**
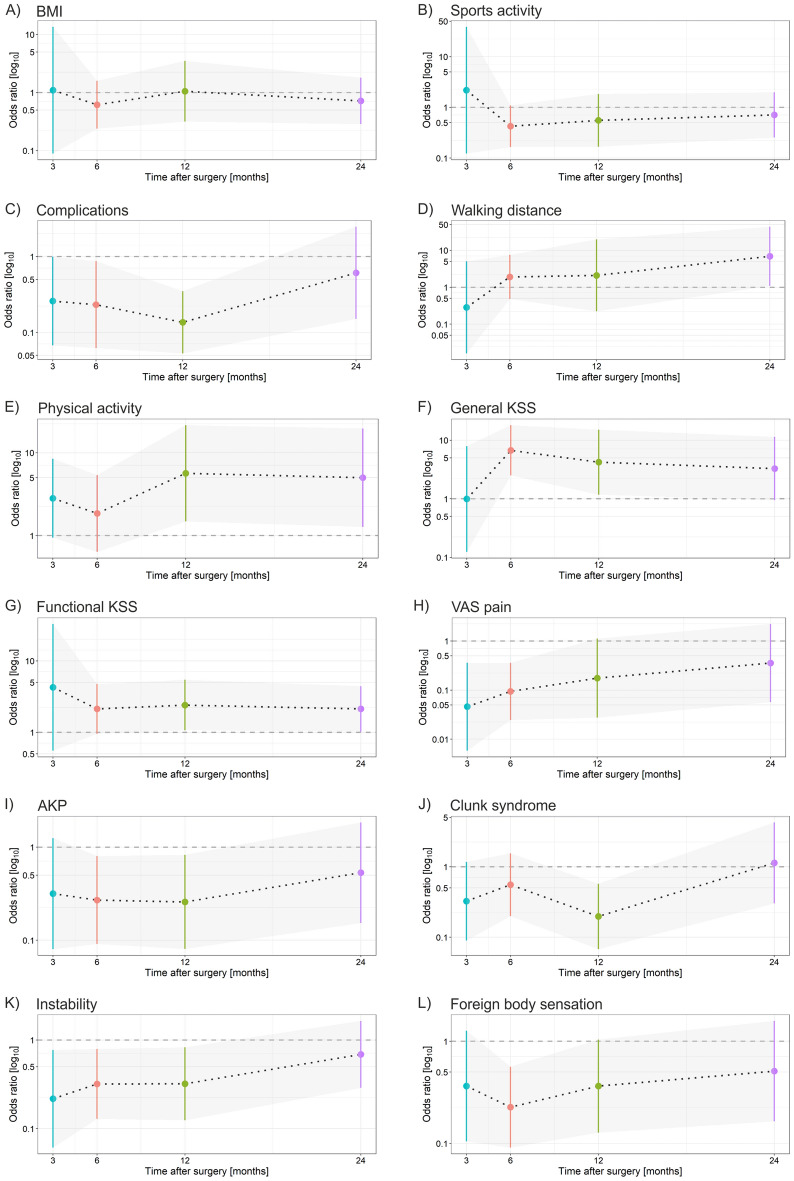
Contribution of postoperative factors to the likelihood of satisfaction in men in the 2 years after TKA. (**A**) BMI, (**B**) sports activity, (**C**) complications, (**D**) walking distance, (**E**) physical activity, (**F**) general KSS, (**G**) functional KSS, (**H**) VAS pain, (**I**) AKP, (**J**) clunk syndrome, (**I**) instability, (**K**) foreign body sensation. Odds-ratio (marked by dots) values above 1 are associated with satisfaction; values below 1 are associated with not being fully satisfied. The bars represent odds ratios (95%CI, low–high value).

In men, the more the general KSS (> 70) increased postoperatively, the higher the likelihood of increased satisfaction compared with those with a lower general KSS. This was more strongly associated with the general KSS 6 and 12 months after surgery (Fig. 2; Table 2). Men with a postoperative functional KSS above 70 had a greater chance of overall satisfaction than men with a lower functional KSS. The functional KSS 3 months after surgery was of little informative value in terms of overall satisfaction (Fig. 2).

#### Postoperative sports activity and physical activities and satisfaction

The positive postoperative factors for satisfaction were physical activity and long-distance walking. In women, these factors were most relevant in relation to overall satisfaction 12 months after surgery (Fig. 1). Walking distance was key to satisfaction during the postoperative check-ups. Women who reported the ability to walk longer distances at postoperative check-ups were more satisfied with the surgery, and the strength of this parameter gradually increased until 12 months after surgery (Fig. 1). In addition, the level of physical activity influenced satisfaction levels in women postoperatively at the 3-month (2.71, 95%CI 1.51–4.85, p < 0.001), 6-month (3.44, 95%CI 1.63–7.26, p < 0.001) and 12-month (4.61, 95%CI 1.75–12.11, p < 0.001) check-ups (Fig. 1; Table 2).

For men, those who reported longer walking distance during check-ups after surgery tended to be more satisfied. In contrast to women, walking distance influenced overall satisfaction with surgery only during the 24-month evaluation (Fig. 2; Table 2). Men who reported more intense activity after TKA were also more satisfied when evaluating the results at 12 (5.65, 95%CI 1.49–21.52, p < 0.001) and 24 months (5.00, 95%CI 1.27–19.66, p = 0.012), but this factor was not statistically significant 3 (2.82, 95%CI 0.94–8.44, p = 0.054) or 6 months (1.85, 95%CI 0.64–5.38, p = 0.253) after TKA surgery (Fig. 2; Table 2).

### Pain and satisfaction

A painful knee postoperatively decreased the chance of overall satisfaction. However, if women reported low VAS pain 3, 6 and 12 months after surgery, they had a greater chance of overall satisfaction with the surgery (Fig. 1; Table 2). Women who did not experience AKP postoperatively had a higher chance of overall satisfaction with the surgery. By contrast, if AKP was reported during postoperative check-ups, the chance of overall satisfaction with the surgery substantially decreased (Fig. 1; Table 2).

If men reported low VAS pain 3, 6 and 12 months after surgery, they had a greater chance of overall satisfaction with the surgery. By contrast, the greater the VAS pain during the check-ups at 3, 6 and 12 months, the lower the overall satisfaction with the surgery (Fig. 2; Table 2). Similar to women, AKP in men functioned as an indicator of lower overall satisfaction with the surgery results. This characteristic was significant 6 and 12 months after surgery. By contrast, if AKP did not occur during postoperative check-ups, the chance of overall satisfaction with the surgery was high (Fig. 2; Table 2).

### Mechanical problems and satisfaction

The analysis revealed a strong association between mechanical symptoms, such as postoperative patellar instability, a foreign body sensation and clunk syndrome, and satisfaction.

If women reported ‘mechanical problems’ during any postoperative check-up in the first 12 months after surgery, their chance of overall satisfaction with the surgery was low. This characteristic was most indicative 6 (0.41, 95%CI 0.18–0.95, p = 0.033) and 12 months (0.31, 95%CI 0.14–0.72, p = 0.005) after surgery. However, clunk syndrome did not influence overall satisfaction with the surgery at 24 months (Fig. 1; Table 2). Women who did not experience instability postoperatively had a higher chance of overall postoperative satisfaction, and vice versa. Instability during check-ups at 3 (0.25, 95%CI 0.13–0.49, p < 0.001), 6 and 12 months was the most indicative (Fig. 1; Table 2). If women reported a foreign body sensation 3 months after surgery, overall satisfaction was less likely (0.38, 95%CI 0.19–0.73, p = 0.003). By contrast, women had a good chance of overall satisfaction if they did not experience this sensation. The association between mechanical problems and satisfaction was not maintained at 24 months, at which time all the women in the study were either satisfied or not fully satisfied irrespective of this phenomenon.

Similar to women, clunk syndrome was also indicative in men. If men reported this characteristic during a check-up, their chance of overall satisfaction with the surgery was lower. This was most indicative during the 12-month postoperative evaluation (0.20, 95%CI 0.07–0.57, p = 0.002). At the check-up 24 months after surgery, clunk syndrome did not influence overall satisfaction with the surgery (Fig. 2; Table 2). If men reported instability during postoperative check-ups, they had a lower chance of overall postoperative satisfaction, similar to women, and vice versa. This was most indicative during the check-ups at 3 (0.22, 95%CI 0.06–0.77, p = 0.010), 6 (0.32, 95%CI 0.13–0.79, p = 0.011) and 12 months (0.32, 95%CI 0.12–0.83, p = 0.015) (Fig. 2; Table 2). Men with a foreign body sensation in the operated knee generally had a lower chance of overall satisfaction with the surgery (Fig. 2). This was most indicative 6 months after surgery (0.23, 95%CI 0.09–0.56, p < 0.001).

### Postoperative complications and satisfaction

The analysis also revealed a relationship between postoperative complications (wound healing disturbances or early reoperation) and overall satisfaction in both sexes. If women experienced a complication, they were less satisfied with the TKA surgery. The critical point for complications was the first year after surgery (0.13, 95%CI 0.06–0.29, p < 0.001); this corresponds with the fact that the frequency of reoperations significantly decreased 1 year after TKA surgery. In men, we also identified a relationship between postoperative complications and satisfaction, particularly if the complications occurred soon after surgery, at 3 (0.26, 95%CI 0.07–0.99, p = 0.036), 6 (0.23, 95%CI 0.06–0.87, p = 0.020) and 12 months (0.14, 95%CI 0.05–0.35, p < 0.001).

### Odds-ratio network analysis: interpretation of visualisation

The visualised network provides a clear and understandable presentation of the analysis results with a high degree of interpretability and explainability. Each cluster (a separate group of similar factors) in the network describes the multivariate outcome of the analysis. To better understand the results, instead of depicting the vertices as circles, icons summarising the essential information from the charts describing the evolution of the ORs in addition to the factor names were used. Furthermore, these icons are coloured (light red for negative, light green for positive and grey for neutral factors), and the icons are framed; the more frames, the stronger (based on the OR value) the role of the factor in overall satisfaction. Thus, in the OR-similarity network, we can identify clusters of factors, which factors are present and, based on tie strengths, the similarity of their longitudinal trends.

In both sexes, three clusters were formed. The right cluster (green) combines factors associated with satisfaction; and the left cluster (red) combines factors associated with not being fully satisfied. The middle cluster for men includes factors (grey) that have little influence on satisfaction (neutral effect); the middle cluster for women includes mostly negative (red) factors, which however have a different trend in relation to satisfaction than the negative factors in the left (red) cluster. The strengths of ties correspond to the similarity between neighbouring vertices (= factors).

**Figure 3:**
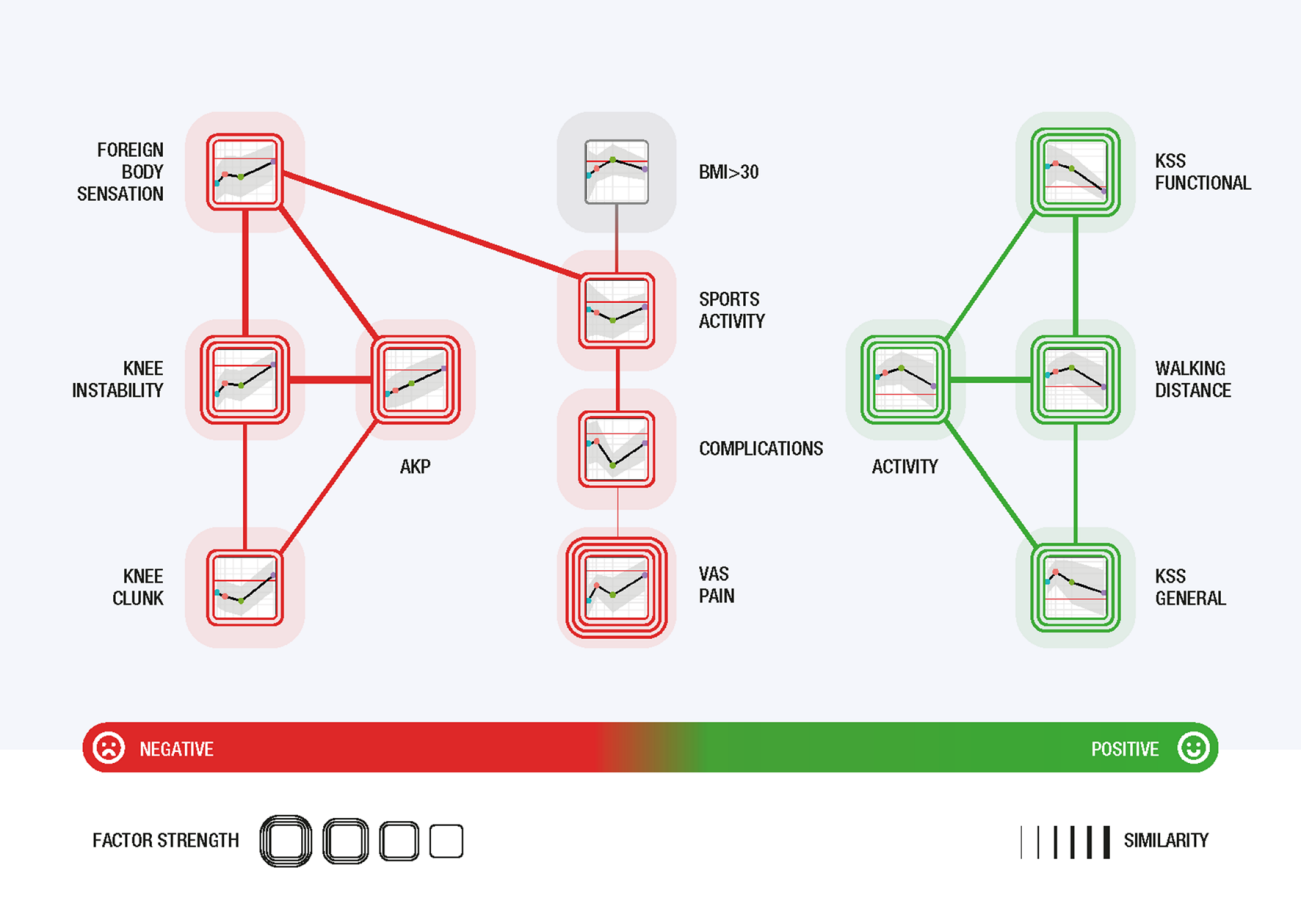
Odds-ratio network for combinations of postoperative factors contributing to satisfaction 2 years after TKA in women. The right cluster (green) combines factors associated with satisfaction, and the left cluster (red) combines factors associated with not being fully satisfied. The size of a network vertex corresponds to its similarity to the neighbouring vertices (= factors).

The negative factor that most strongly influenced women to not be fully satisfied was VAS pain, followed by AKP with knee instability and then other mechanical problems (clunk syndrome and foreign body sensation), complications and low sports activity. Satisfaction in women was strongly associated with activity and long-distance walking, followed by function characteristics of the knee (general and functional KSS). BMI was identified as a neutral factor and was not associated with satisfaction in women (Fig. 3).

**Figure 4:**
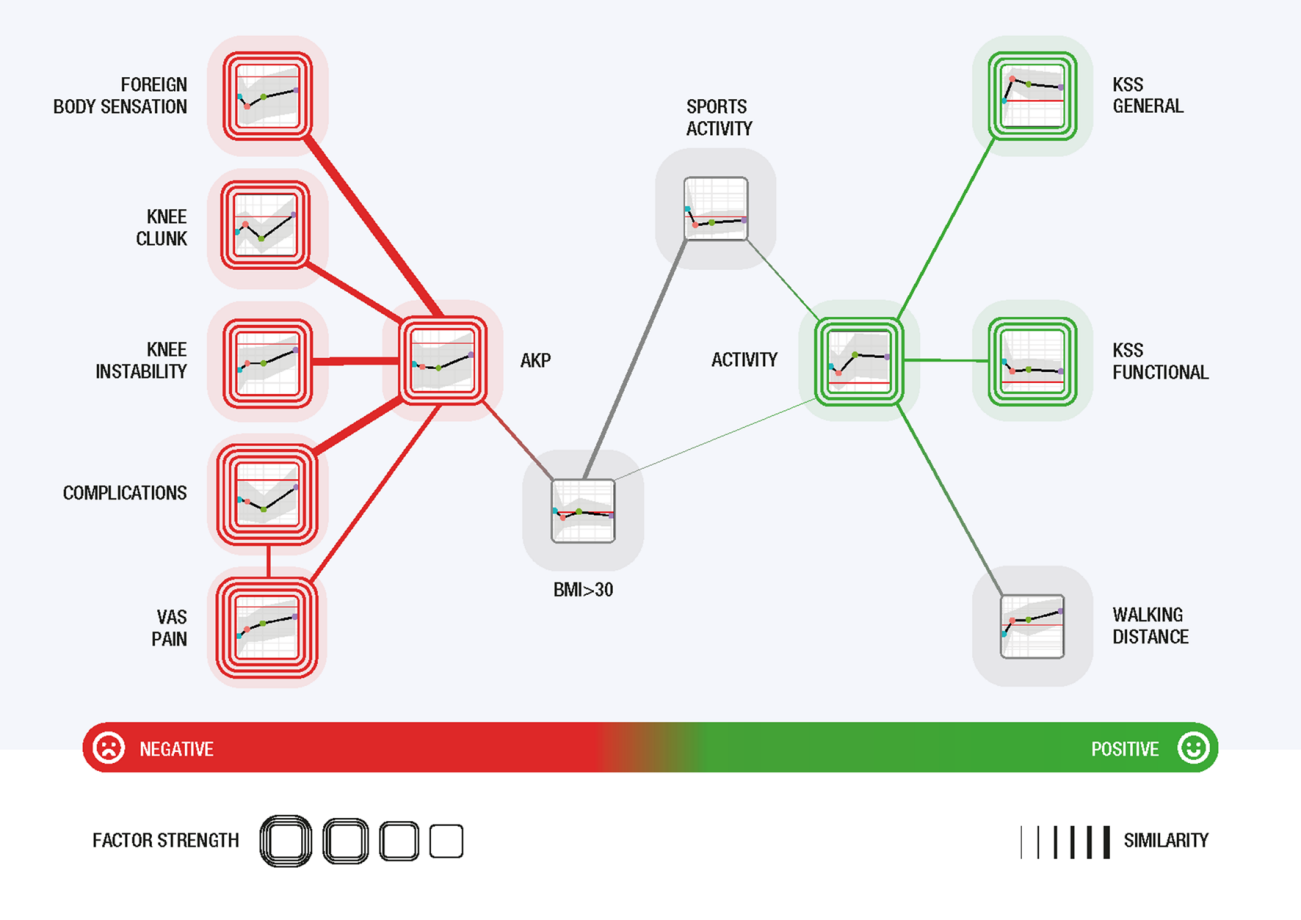
Odds-ratio network for combinations of postoperative factors contributing to satisfaction 2 years after TKA in men. The right cluster (green) combines factors associated with satisfaction, the middle cluster network (grey) includes factors that have little influence on satisfaction (neutral effect) and the left cluster (red) combines factors associated with not being fully satisfied. The size of a network vertex corresponds to its similarity to the neighbouring vertices (= factors).

In men, the strongest negative factors regarding satisfaction were VAS pain and complications, followed by AKP and mechanical problems (knee instability, foreign body sensation and clunk syndrome). The most representative positive factor associated with satisfaction in men was activity, followed by general KSS, functional KSS and the ability to walk long distances (Fig. 4).

In both sexes, the 3-month check-up after TKA was key to the assessment of satisfaction in the 2 years after TKA, whereas a check-up at 6 months was less indicative.

## Discussion

This study investigated the relationship between a set of factors and satisfaction with TKA, revealing that postoperative factors contribute differently to satisfaction in women and men during the first 2 years after surgery. The 3-month check-up provided the most information regarding satisfaction, a key outcome measure of TKA.

Assuming both pain relief and increase in function are delivered by TKA, satisfaction should increase after surgery. However, up to 28% of patients are not fully satisfied with their TKA surgery, regardless of their clinical or radiographic findings^1–5^. Therefore, research on satisfaction-related factors and their combinations is warranted. However, obtaining relevant information from individual patients regarding satisfaction is challenging because it is a multifactorial and psychologically complex term combining health factors, non-health experiences and feelings^7,19^. As a result, heterogenous methods for measuring satisfaction are generally used^2^. Moreover, patient self-evaluation of satisfaction depends not only on the patient’s reading and understanding of the questions on satisfaction but also on the question number^20^ and communication linked to examinations^21^. In addition, according to Herzberg’s motivational hygiene theory, patients are often unable to understand that satisfaction and dissatisfaction are not opposites^22^.

To minimise the influence of misleading responses about satisfaction by individuals, this study focused on the trend analysis of factors related to satisfaction among groups of women and men. Moreover, the OR for each factor associated with satisfaction is presented with 95%CI, a range of values that refers to the probability that a single leading value will fall between two set values. In our real-world cohort, persistent pain and mechanical symptoms were among the most significant negative factors affecting satisfaction. Regarding pain, its negative link to satisfaction is expected and has been reported by numerous studies^1,11^. Chronic pain after TKA is an additional very strong multifactorial problem that is still waiting for a definitive solution^23^. In general, patients should be informed about possible pain even after a well-performed TKA, and patients at risk of pain should be identified early and treated intensively. Additionally, in our patients, postoperative mechanical symptoms and complications requiring reoperations decreased satisfaction with TKA, which is consistent with the findings of other studies^24–27^. Based on our experience, all potential sources negatively influencing satisfaction should be explained in detail to a patient at the regular check-ups. Minor symptoms should be treated using intensive physiotherapy, followed by a continuous home programme and psychological support. Major symptoms related directly to the surgery, such as knee instability and patella-related pain, should be resolved during early reoperation. For both women and men, the most positive factors associated with satisfaction were physical activity and higher general and functional KSS soon after TKA. Associating rapidly growing functional capacity after TKA with painless and stable TKA in patients who are competent in terms of psycho–neuro–motor activity seems reasonable.

Our data and the findings of other studies^1^ suggest that a series of possibly interrelated factors rather than a single leading factor contribute to satisfaction after TKA. To provide further insights into this satisfaction, we constructed OR-similarity networks based on the longitudinal trends in ORs of analysed factors in relation to satisfaction during the first 2 years after TKA. Our multivariate analysis revealed different patterns in factors contributing to satisfaction in women and men. In men, the strongest negative factors were VAS pain and complications, followed by AKP and mechanical problems (instability, foreign body sensation and clunk syndrome). The negative factors strongly influencing satisfaction in women were VAS pain, followed by AKP with knee instability, other mechanical problems, complications and low sports activity. In line with another report^28^, physical activity and general and functional KSS predisposed both sexes to satisfaction; long-distance walking was associated with satisfaction only in women.

Establishing when the outcome after TKA surgery is sufficiently stabilised to evaluate final satisfaction levels is also clinically important. Our data reveal that the key check-up for assessing the evolution of satisfaction was 3 months after TKA in both sexes. A recent study of 86 patients demonstrated that patients with a poor satisfaction trajectory can be detected as early as 6 weeks postoperatively, and satisfaction scores significantly improve within the first 6 months after TKA and subsequently stabilised^12^. In our study, we were unable to evaluate the satisfaction scores earlier because most of our patients were still undergoing complex and long-term physiotherapy 6 weeks after TKA. However, our data further support the importance of early check-ups after TKA because they have the highest relevance for the development of final satisfaction. Moreover, an early identification that patients are not fully satisfied may help address their problems through intensive and focused physiotherapy, pharmacological and psychological support and/or early reoperation.

Our longitudinal trend study also revealed that patient satisfaction changes substantially during the 2 years after TKA, with significant differences between women and men in the process of acceptance of TKA. If men were not fully satisfied at postoperative month 3 or 6, their chances of overall satisfaction with TKA at 2 years decreased. By contrast, women who were not fully satisfied soon after TKA were just as likely to be satisfied 2 years postoperatively as those who reported satisfaction after 3–6 months. However, women and men reporting physical activity early after TKA surgery had an increased chance of final satisfaction compared with patients with low activity levels. The gender-related differences observed in our study cannot be easily explained. Women may be more realistic about the final outcome of TKA, and they might also adapt more easily to not being fully satisfied than men. However, women may also choose a softer and more differentiated approach to their evaluation, hesitating more with a choice signalling lack of satisfaction.

This study has several strengths. Visualising time-dependent changes in factors related to satisfaction makes it possible to track the trend of individual factors and compare them with each other. We applied standard statistical approaches, and by visualising the data using networks, the data might be interpreted as more complex, with remaining interpretable and explainable outputs for clinicians and patients. This multivariate analysis better reflects the contribution of multiple parameters of various origins (clinics–demographics–expectations–feelings) to clinical outcomes and patient satisfaction and its development over time. This study also has several limitations. First, our results are based on a single local TKA registry, and future studies should verify our results. Second, some of the patients attended check-ups at outpatient departments outside our clinic, resulting in data incompleteness, but the analysis of data from groups of women and men, including CIs, addressed these missing values.

## Conclusions

Satisfaction after TKA in the first 2 years after surgery reveals different patterns in women and men. The OR-based visualisation used, including CIs, helped explain and interpret the relationships between combinations of factors underlying satisfaction. Assessing the causal factors contributing to patients not being fully satisfied with TKA may enable the formulation of effective strategies to improve satisfaction with TKA.

## Data Availability

Data are available from the the corresponding author upon reasonable request and with permission of the University Hospital Olomouc.
